# *Arc* expression identifies the lateral amygdala fear memory trace

**DOI:** 10.1038/mp.2015.18

**Published:** 2015-03-24

**Authors:** L A Gouty-Colomer, B Hosseini, I M Marcelo, J Schreiber, D E Slump, S Yamaguchi, A R Houweling, D Jaarsma, Y Elgersma, S A Kushner

**Affiliations:** 1 Department of Psychiatry, Erasmus University Medical Center, Rotterdam, The Netherlands; 2 Champalimaud Neuroscience Programme, Champalimaud Centre for the Unknown, Lisbon, Portugal; 3 Department of Neuroscience, Erasmus Medical Centre, Rotterdam, The Netherlands; 4 Division of Morphological Neuroscience, Gifu University Graduate School of Medicine, Gifu, Japan; 5 PRESTO, Japan Science and Technology Agency (JST), Saitama, Japan

## Abstract

Memories are encoded within sparsely distributed neuronal ensembles. However, the defining cellular properties of neurons within a memory trace remain incompletely understood. Using a fluorescence-based *Arc* reporter, we were able to visually identify the distinct subset of lateral amygdala (LA) neurons activated during auditory fear conditioning. We found that *Arc-*expressing neurons have enhanced intrinsic excitability and are preferentially recruited into newly encoded memory traces. Furthermore, synaptic potentiation of thalamic inputs to the LA during fear conditioning is learning-specific, postsynaptically mediated and highly localized to *Arc*-expressing neurons. Taken together, our findings validate the immediate-early gene *Arc* as a molecular marker for the LA neuronal ensemble recruited during fear learning. Moreover, these results establish a model of fear memory formation in which intrinsic excitability determines neuronal selection, whereas learning-related encoding is governed by synaptic plasticity.

## Introduction

Fear conditioning is a robust form of associative learning in which a previously neutral conditioned stimulus (CS) comes to predict an aversive unconditioned event, eliciting defensive behaviors and fearful emotions.^1^ The neurobiological circuitry underlying auditory fear learning has been extensively investigated, wherein there is overwhelming evidence that the lateral amygdala (LA) is a critical site of plasticity.^2, 3^ In particular, long-term *N*-methyl-_D_-aspartate (NMDA) receptor-dependent synaptic potentiation of glutamatergic inputs onto LA principal neurons remains the leading candidate mechanism for fear memory encoding.^4^ Accordingly, both genetic and pharmacological blockade of synaptic plasticity in the LA prevent the formation of long-term fear memories,^5, 6, 7, 8, 9^ whereas potentiation of glutamatergic synaptic transmission onto LA pyramidal neurons is induced by fear conditioning.^5, 6, 10, 11, 12^

Intriguingly, only a limited subset of neurons appears to be recruited during fear memory encoding. In particular, recent studies have implicated the cAMP response element-binding protein (CREB) as a critical factor guiding LA neuron recruitment into a fear memory network. Targeted restoration of CREB expression selectively into the LA of CREB-deficient mice is sufficient to fully restore auditory fear conditioning.^13^ Furthermore, optogenetic activation of neurons with elevated CREB levels at the time of training is sufficient to induce fear memory retrieval.^14^ Moreover, studies using virus-mediated mosaic overexpression of CREB in wild-type mice have shown that recruitment of LA neurons during fear learning is not merely a cell autonomous process,^13^ but rather is dependent upon relative neuronal excitability at the time of learning.^15, 16^ Importantly, however, the hypothesis that the recruitment of LA neurons into fear memory networks is determined by their relative excitability has never been evaluated under endogenous physiological conditions.

Recent computational modeling has proposed that the encoding of fear memories in the LA is constrained to a limited subset of neurons by the local microcircuitry through a combination of intrinsic excitability and synaptic plasticity.^17^ Consistent with this model, *in vivo* extracellular single-unit recordings have demonstrated that only a minority of LA neurons undergo significant changes in tone-evoked firing during auditory fear conditioning.^18, 19^ Furthermore, *ex vivo* whole-cell patch-clamp recordings also found that learning-induced plasticity was restricted to a limited subset of LA neurons.^5^

Recent studies have provided strong experimental support that immediate-early genes (IEGs), including the proto-oncogene *c-Fos* and the activity-regulated cytoskeleton-associated protein (*Arc*), represent time-limited molecular tags of these sparsely encoded neurons in both sensory representations^20, 21, 22^ and memory networks.^13, 23, 24, 25, 26, 27^ Inhibition or ablation of IEG-tagged neurons disrupts the recall and maintenance of fear-conditioning memories, respectively.^15, 28^ Conversely, artificial activation of this sparse IEG-tagged population is sufficient to induce fear memory recall^29^ or falsely modify contextual memories.^30, 31^ Importantly, however, no previous studies have performed targeted electrophysiological recordings from a defined memory trace, a crucial step towards achieving a comprehensive understanding of how the brain encodes learned associations.

Therefore, to investigate the neurophysiological properties of individual LA neurons recruited during fear conditioning, we utilized a fluorescence-based reporter of *Arc* as a time-limited molecular tag of these sparsely encoded neurons. We found that neurons with elevated baseline intrinsic excitability were preferentially recruited into the fear memory network. Furthermore, synaptic potentiation of thalamic inputs to the LA during fear conditioning was learning-specific and highly localized to *Arc*-expressing neurons. Taken together, our findings establish a model of fear memory formation in which intrinsic excitability determines neuronal selection, whereas learning-related encoding is governed by synaptic plasticity.

## Materials and methods

### Animals

*Arc::dVenus* mice were backcrossed more than 10 generations into C57BL/6J.^32^ Mice were maintained on a 12 h light/dark cycle with food and water available *ad libitum*. All experiments were performed during the light phase, using adult mice (postnatal weeks 8–11). Mice were individually housed for 5 days prior to the start of experiments. Randomization was assigned based on the outcome of the littermate genotyping, and experimenter blinding was performed whenever possible. All experiments were approved by the Dutch Ethical Committee and in accordance with the Institutional Animal Care and Use Committee (IACUC) guidelines.

### Auditory fear conditioning

Fear conditioning was performed using a Med Associates Standard Fear Conditioning chamber (30.5 cm × 24.1 cm × 21.0 cm) with a stainless steel electrifiable grid floor, and enclosed within a larger sound-attenuating box. Video images were recorded using a progressive scan CCD video camera with a visible light filter suitable for near-infrared imaging. Mice in the naïve group received no handling or exposure to the training context. Naïve mice remained in their standard housing conditions until immediately prior to behavioral testing for CS-evoked freezing, perfusion for confocal imaging or killing for electrophysiology. In contrast, mice in the unpaired and paired training groups were habituated to the conditioning chamber, 24 h prior the training session. Habituation sessions consisted of a 30 min exposure to the training context without any tone or shock presentations. On the day of conditioning, mice receiving paired training were placed in the conditioning chamber for 180 s, followed by a series of three co-terminating presentations of a tone CS (30 s, 5 KHz, 85 dB) and scrambled footshock unconditioned stimulus (US) (2 s, 0.75 mA). The intertrial interval between tone-shock presentations was 210 s. The experiments shown in Supplementary Figure 2 comparing the strength of conditioning and *Arc*-dVenus activation following 1, 3 or 9 CS-US pairings used independent groups of mice. Training was implemented using the same parameters (180 s placement-to-shock interval, 210 s interstimulus interval) and CS/US stimuli as the paired condition. Mice in the unpaired group received the identical CS and US stimuli but in an explicitly unpaired sequence. The unpaired protocol consisted of 3 US presentations (10 s interstimulus interval) in which the first shock was delivered immediately upon placement in the chamber, and followed by 3 CS presentations initiated 400 s after the last US presentation (90 s interstimulus interval). Previous studies using similar explicitly unpaired controls have demonstrated that subjects acquire minimal or no associative fear of the CS.^1, 33^ Tone-evoked freezing was tested 24 h after conditioning in a novel context (120 s baseline, 180 s tone). Freezing was defined as the cessation of all movement except for respiration and scored using an automated algorithm.^34^

### Immunofluorescence

After deep anesthesia induced by intra-peritoneal injection of pentobarbital (50 mg kg^−1^), mice were transcardially perfused with saline, followed by 4% paraformaldehyde. Brains were dissected and post-fixed in 4% paraformaldehyde for 2 h at 4 °C. After post-fixation, the brains were transferred into 10% sucrose phosphate buffer (PB 0.1 M, pH 7.3) and stored overnight at 4 °C. Embedding was performed in a 10% gelatin+10% sucrose block, with fixation in 10% paraformaldehyde+30% sucrose solution for 2 h at room temperature and immersed in 30% sucrose at 4 °C. Forty micrometer coronal sections were collected serially (rostral to caudal) using a freezing microtome (Leica, Wetzlar, Germany; SM 2000R) and stored in 0.1 M PB. Free-floating sections were incubated in sodium citrate (10 mM) at 80 °C for 1 h and rinsed with tris-buffered saline (TBS, pH 7.6). Sections were pre-incubated with a blocking TBS buffer containing 0.5% Triton X-100 and 10% normal horse serum (NHS; Invitrogen, Bleiswijk, The Netherlands) for 1 h at room temperature. Sections were incubated in a mixture of primary antibodies, in TBS buffer containing 0.4% Triton X-100 and 2% NHS for 72 h at 4 °C.

The following primary antibodies were used: mouse anti-NeuN (1:2000, Millipore, Hertfordshire, UK; MAB377), goat anti-choline acetyltransferase (1:200, Millipore AB144P), rabbit anti-Tbr1 (1:2000, Millipore AB10554), mouse anti-Arc (C-7, 1:200, Santa Cruz sc-17839, Heidelberg, Germany), rabbit anti-c-Fos (ab-5, 1:10000, Millipore PC38), mouse anti-GAD67 (1:1000, Millipore MAB5406). Sections were washed with TBS, and incubated with corresponding Alexa-conjugated secondary antibodies (1:200, Invitrogen) and cyanine dyes (1:200, Sanbio, Uden, The Netherlands) in TBS buffer containing 0.4% Triton X-100, 2% NHS for 2 h at room temperature. For some experiments, nuclear staining was performed using DAPI (1:100, Invitrogen). Sections were washed with PB 0.1 M and mounted on slides, cover slipped with Vectashield H1000 fluorescent mounting medium (Vector Labs, Peterborough, UK), and sealed.

### Fluorescent *in situ* hybridization

Fluorescent *in situ* hybridization was performed using mice perfused at 5 min post training, to optimally visualize nuclear foci of *dVenus* and *Arc* transcription.^23^ Coronal brain sections (40 μm) were collected in RNAse-free 0.1 M PB as described in the immunofluorescence section. The cDNA templates encoding the following mRNAs were used for single-stranded RNA probe synthesis: *Arc/Arg3.1* (3.5 kb, full length probe, GeneID: 11838; Image Clone number: 349057; generously provided by J. Holstege and M. Hosseini); *Venus fluorescent protein* (720 kb probe from pISH-Venus Addgene plasmid 15865, kindly deposited by P. Mombaerts). The riboprobes were obtained by linearizing the recombinant plasmids with the appropriate restriction enzymes (Fermentas, Bleiswijk, The Netherlands; New England BioLabs, Hitchin, UK) and RNA polymerases. Transcription was performed in the presence of digoxigenin or fluorescein-labeled 11-UTP (Roche, Almere, The Netherlands), for *Venus* or *Arc* riboprobes, respectively, using a commercial RNA labeling kit (Roche). Riboprobes were purified by standard LiCl precipitation protocol. Integrity and yield of riboprobes was confirmed by gel electrophoresis and Nanodrop spectrophotometry (Thermo Scientific, Waltham, MA, USA). All solutions used until the completion of hybridization were treated with Diethylpyrocarbonat to ensure optimal RNAse-free working conditions.

The protocol used for fluorescent *in situ* hybridization was adapted from Hossaini *et al.*^35^ Free-floating sections were first washed in 0.1 M PB, treated for 5 min with 0.2% glycine in PBS, rinsed in PBS and fixed for 10 min in 4% paraformaldehyde. After another rinse in PBS, sections were treated (10 min) in PBS containing 0.1 M triethanolamine (Merck, Hertfordshire, UK) pH 8.0 and 0.0025% acetic anhydride (Sigma-Aldrich, Munich, Germany). Sections were then washed in 4 × standard saline citrate (pH 4.5) and prehybridized for 1 h at 65 °C in hybridization solution consisting of 5 × standard saline citrate (pH 4.5), 50% formamide (Sigma-Aldrich), 2% Blocking Reagent (Roche), 0.05% 3-[(3-cholamidopropyl)dimethylammonio]-1-propanesulfonate (Sigma-Aldrich), 1 μg ml^−1^ yeast tRNA (tRNA brewer's yeast, Sigma-Aldrich), 50 μg ml^−1^ Heparin (Sigma-Aldrich) and 5 mM ethylenediaminetetraacetic acid (pH 8.0, Sigma-Aldrich). Sections were hybridized for 18–24 h at 65 °C in hybridization solution containing 1.2 μg ml^−1^ of each anti-sense riboprobe, *Arc/Arg3.1* and *Venus*. After hybridization, sections were washed in 2 × standard saline citrate (pH 4.5), followed by three washes of 15 min at 65 °C in 2 × standard saline citrate (pH 4.5) and 50% formamide, and a final wash in PBS. The sections were then pre-incubated for 90 min at room temperature in blocking buffer, consisting of 0.5% Triton X-100 and 10% NHS in TBS. For detection of the digoxigenin and fluorescein tags in riboprobes, sections were incubated in 0.4% Triton X-100 and 2% NHS in TBS (pH 7.6), with primary sheep polyclonal anti-digoxigenin (1:500, Thermo Scientific) and mouse monoclonal anti-fluorescein (1:500, Roche) antibodies, for 72 h at 4 °C. Subsequently, sections were washed with TBS and detection of anti-digoxigenin and anti-fluorescein primary antibodies was carried out using anti-sheep Cy3 from donkey (1:200, Jackson Laboratories, Bar Harbor, ME, USA) and anti-mouse Alexa647 from donkey (1:200, Jackson Laboratories), respectively, in 0.4% Triton X-100 and 2% NHS in TBS (pH 7.6) at room temperature, for 2 h. Sections were then washed in 0.1 M PB and stained using DAPI (1:100, Invitrogen) as a nuclear marker. Sections were then mounted on slides, cover slipped with Vectashield H1000 fluorescent mounting medium (Vector Labs) and sealed.

### Confocal imaging

Stained LA images were acquired using a Zeiss LSM 700 confocal microscope (Carl Zeiss, Oberkochen, Germany) equipped with Zeiss Plan-Apochromat × 10/0.45, × 20/0.8 and × 40/1.3 (oil immersion) objectives. Native dVenus, Cy3, Alexa647 and DAPI were imaged using the excitation wavelengths of 488, 555, 639 and 405 nm, respectively. Native dVenus fluorescence intensity was quantified using ImageJ (NIH, 1.42q) with the Multi Measure plug-in. The mean fluorescence intensity of each *Arc-*dVenus^+^ neuron was determined by drawing a region of interest around the cell soma.

### Stereology

Coronal brain sections were collected serially through the entire extent of the LA of each mouse, with a section thickness of 40 μm and interval distance of 160 μm (Supplementary Figure 1). Sections were immunofluorescently labeled with anti-NeuN and anti-choline acetyltransferase to identify mature neurons and to define the border between the lateral and basolateral nuclei of the amygdala,^36^ respectively. Furthermore, to optimally standardize the stereological analysis and in light of recent findings demonstrating hemispheric lateralization of Arc expression within the insular cortex following taste learning,^37^ all stereological and fluorescence intensity data were collected exclusively from the left hemisphere.

Stereological estimation of the total population (NeuN^**+**^) and Arc-dVenus^**+**^ subset of neurons was performed using the Optical Fractionator probe within Stereo Investigator (version 10, MBF Bioscience, Williston, VT, USA). Stacks of confocal images (156 × 156 × 1 μm) across the thickness of the sections (with a separation level of 1 μm) of Arc-dVenus^**+**^ and NeuN^**+**^ neurons were systematically collected. A counting frame size of 100 μm × 100 μm was used to mark Arc-dVenus^**+**^ neurons throughout the entire grid, using an exhaustive sampling configuration. NeuN^**+**^ cells were counted using 35 μm × 35 μm counting frames, which were selected in a systematic random procedure by the analysis software. The grid size of both exhaustive and random sampling configurations was set to 100 μm × 100 μm.

The section thickness was assessed empirically at every sampling site to precisely calculate any potential thickness variation across the sections as a result of post-processing of the tissue. Guard zones (2 μm) were used at the top and bottom of each section with a dissector height of 15 μm. Accuracy in the estimation of the total number of quantified cells per subject was estimated using the coefficient of error equations.^38, 39, 40^ Coefficient of error values were <0.1 in all mice analyzed.

### Brain slice electrophysiology

Mice were anesthetized using isoflurane, decapitated and the brain dissected in ice-cold modified artificial cerebrospinal fluid (ACSF) containing the following (in mM): 110 NaCl, 2.5 KCl, 2 CaCl_2_, 2 MgCl_2_, 1 NaH_2_PO_4_, 25 NaHCO_3_, 10 glucose, 0.2 ascorbate, 0.2 thiourea. Acute coronal slices (300 μm) containing the LA were cut using a vibratome (Microm 650 V, Thermo Scientific) and transferred to a storage chamber in ACSF, saturated with 95% O_2_/5% CO_2_ and maintained at 32–34 °C. After at least 1 h of recovery time, slices were transferred to the recording chamber where they were continuously perfused with oxygenated ACSF at a perfusion rate of 1.5–2 ml min^−1^.

Whole-cell patch-clamp recordings of LA neurons were performed at 32–34 °C under infrared differential interference contrast visual guidance using an upright microscope (Nikon, Tokyo, Japan; Eclipse E600FN). *Arc*-dVenus^+^ fluorescence cells were detected *via* illumination of a mercury lamp using a YFP filter (Semrock, Rochester, NY, USA). Borosilicate glass pipettes (4–7 MOhm) were connected to an Axon Multiclamp 700B amplifier (Molecular Devices, Sunnyvale, CA, USA) and data were acquired at 20 KHz, filtered at 3 KHz, stored and analyzed using the pClamp software (pClamp 10, Molecular Devices). Pipettes were filled with the following medium (in mM): 130 KMeSO_3_, 11 KCl, 10 HEPES, 5 NaCl, 0.1 EGTA, 1 MgCl_2_, 2 Mg-ATP, 0.3 Na-GTP, 5 phosphocreatine, 50 U ml^−1^ creatin phosphokinase, the pH was adjusted to 7.2 and osmolarity to 290 mOsm. Slices were continuously superfused with ACSF, saturated with 95% O_2_/5% CO_2_ and maintained at 32–34 °C. Liquid junction potential was left uncorrected. Except for measurements of intrinsic properties, the GABA_A_ receptor blocker picrotoxin (00 μM) was added to the ACSF. Large, pyramidal-like somata were visualized targeted for recordings, and readily distinguished from fast-spiking neurons, characteristic of LA interneurons.^41, 42, 43^ No fast-spiking neurons were found in the *Arc*-dVenus^**+**^ population, consistent with *Arc* expression in the LA being limited to glutamatergic principal neurons.^44^

Passive membrane properties were analyzed using a 10 mV hyperpolarizing voltage step in voltage-clamp mode. Resting membrane potential was measured immediately after establishing the whole-cell configuration. Single action potentials (APs) were evoked by a 10 ms current injection whose amplitude was minimally sufficient to reach the threshold from a potential of −75 mV. The threshold was defined as the inflection point at the foot of the regenerative upstroke. AP amplitude and after-hyperpolarizing potential were measured from the threshold to the peak and to the maximal hyperpolarizing value, respectively. AP duration was measured at half of the maximal amplitude.

For evoked postsynaptic currents, thalamic fibers of the ventral part of the striatum were stimulated using a bipolar Platinum-Iridium electrode (FHC, Bowdoin, ME, USA). Postsynaptic responses were recorded from *Arc*-dVenus^**−**^ and *Arc*-dVenus^+^ neighboring cells, thereby reducing interslice variability. Input-output curves were constructed by varying the stimulus intensity from 0 to 200 μA (in 25 μA increments) at 0.1 Hz. Excitatory postsynaptic current (EPSC) amplitude was normalized by the cell capacitance. Paired-pulse ratio was analyzed as the ratio of the second to the first EPSC resulting from two consecutive stimulations, in which the interstimulus interval ranged from 25 to 100 ms (in 15 ms increments) and from 100 to 300 ms (in 25 ms increments). For α-amino-3-hydroxy-5-methyl-4-isoxazolepropionic acid (AMPA)/NMDA recordings, the intracellular solution was modified by substituting KMeSO_3_ and KCl with CsMeSO_3_ and CsCl, respectively. The AMPA component was measured as the peak current recorded at −70 mV. The NMDA component was recorded at +40 mV (measured 100 ms after stimulus onset), and entirely blocked in the presence of 1-amino-phosphovaleric acid (50 μM).

### Statistical analysis

Significance of observations was established by unpaired Student's *t* test or analysis of variance followed by Tukey's *post hoc* test. Cumulative probability distributions of fluorescence intensity were compared using the Kolmogorov–Smirnov test. Data are expressed as mean±s.e.m. Significance threshold was set at *P<*0.05 for all statistical comparisons.

## Results

### *Arc-*dVenus expression accurately reflects endogenous *Arc* transcription

**Figure 1:**
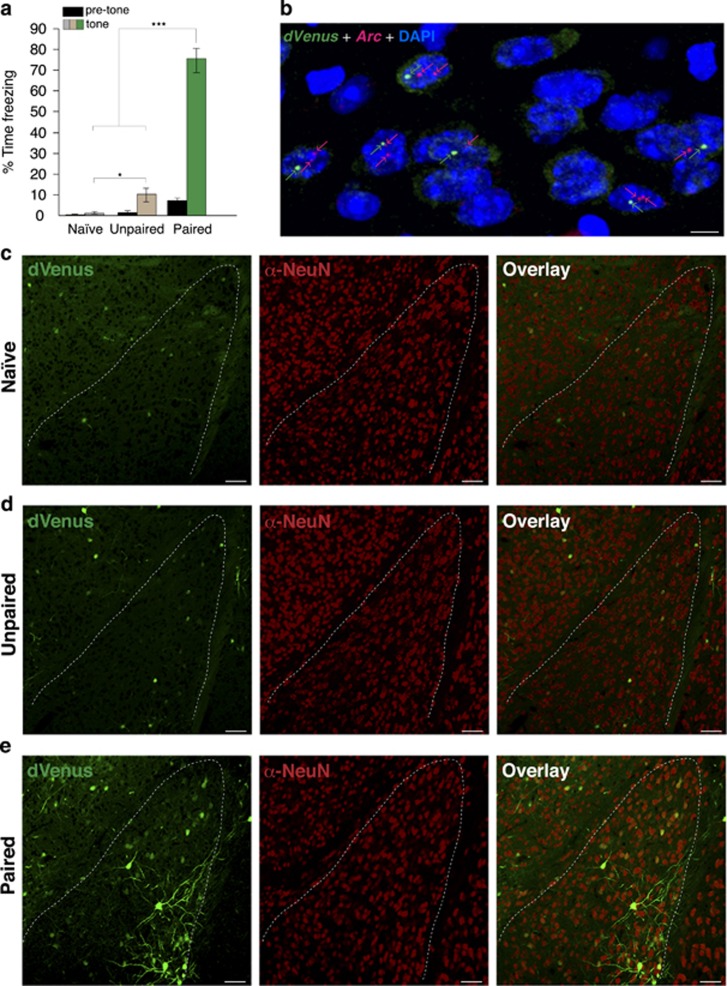

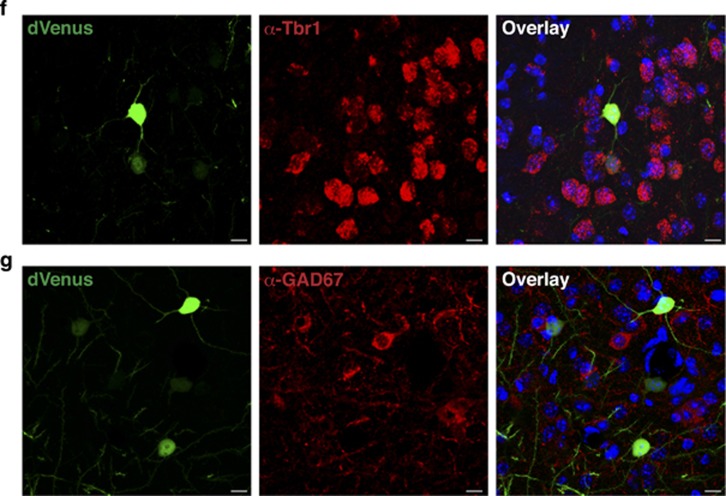
Fear conditioning induces learning-specific activation of *Arc*-dVenus in the lateral amygdala (LA). (**a**) Tone-induced freezing in naïve mice, and those receiving either paired or unpaired presentations of tone and shock (*n*=8 mice/group). One-way analysis of variance, *F*=107.07, *P<*0.001. *P<0.05, ***P<0.001. (**b**) *Arc-dVenus* reporter and endogenous *Arc* RNA intra-nuclear foci (indicated by arrows) are highly co-localized. Scale bar, 5 μm. (**c**–**e**) *Arc*-dVenus^+^ expression in the LA of mice from naïve (**c**), unpaired (**d**) and paired (**e**) conditions killed 5 h post training. Dotted lines indicate LA boundaries. Scale bar, 50 μm. (**f** and **g**) Matching the cell-type specificity of endogenous *Arc* expression, *Arc*-dVenus^+^ neurons in the LA uniformly express the glutamatergic marker Tbr1 (*n*=1900 cells; F), but not the GABAergic marker GAD67 (*n*=1140 cells; G). Scale bar, 10 μm.

To visualize LA neurons recruited during fear learning, we utilized a recently engineered mouse line expressing destabilized Venus fluorescent protein (dVenus) under the control of a transgenic *Arc* promoter (*Arc::dVenus* mice), thereby leaving the endogenous *Arc* genes unmodified.^32^ Hence, these mice function as a fluorescence-based reporter of *Arc* transcription without interfering with the function of endogenous *Arc* itself. Using compartmental analysis of temporal gene transcription by fluorescence *in situ* hybridization,^23^ we confirmed the high co-localization of *Arc*-*dVenus* and endogenous *Arc* nuclear RNA in the LA after auditory fear conditioning, thereby demonstrating the validity of *Arc::dVenus* reporter mice for visualizing LA cells with recent endogenous *Arc* activation (Figure 1b).

To examine the specificity of *Arc*-dVenus activation during fear learning, we used three independent groups: naïve (homecage) controls, explicitly unpaired presentations of tone and shock, or paired tone-shock conditioning (Figure 1a). Mice were killed 5 h after fear conditioning, consistent with previous reports demonstrating that maximal experience-driven *Arc*-dVenus expression occurs within 4–6 h.^32, 45^ *Arc*-dVenus fluorescence was robustly increased in the LA of mice receiving paired training (Figure 1e). In contrast, naïve mice (Figure 1c) and those receiving unpaired training (Figures 1d) showed relatively weaker fluorescence, confirming the specificity of *Arc*-dVenus activation in the LA to fear learning.

Previous studies have demonstrated that endogenous *Arc* expression is localized to principal neurons within the forebrain.^44^ Therefore, to confirm the cell-type specificity of the *Arc*-dVenus reporter in the LA, we performed immunohistochemical labeling with antibodies against Tbr1 or GAD67, markers for glutamatergic projection neurons or GABAergic interneurons, respectively.^46^ Indeed, we found that *Arc*-dVenus^**+**^ neurons in the LA were always NeuN^**+**^ (Figures 1c–e) and Tbr1^**+**^ (Figure 1f). Conversely, we never observed an *Arc*-dVenus^**+**^ neuron that was GAD67^**+**^ (Figure 1g), thereby confirming that *Arc*-dVenus expression is exclusively limited to glutamatergic neurons in the LA, consistent with the cell-type specificity of endogenous *Arc*.

### Fear learning robustly and selectively induces *Arc-*dVenus expression

**Figure 2:**
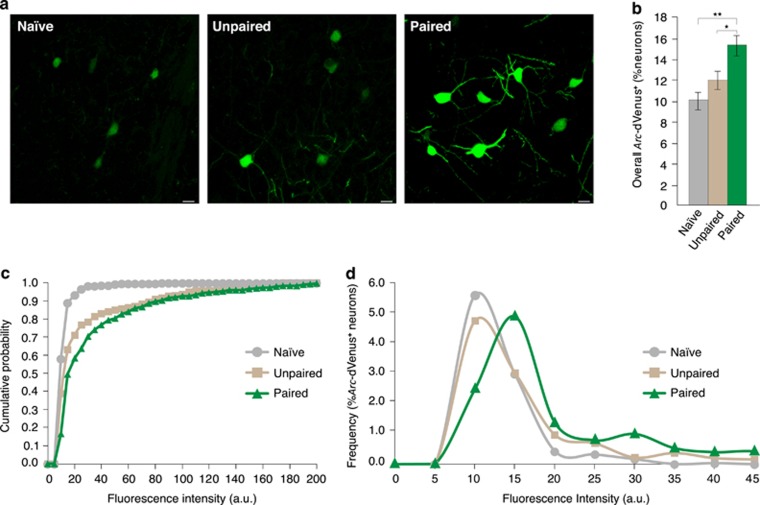
*Arc*-dVenus expression is selectively induced by fear learning. (**a**) Native dVenus fluorescence from naïve, unpaired and paired mice at 5 h post training. Scale bar, 10 μm. (**b**) Stereological quantification of *Arc*-dVenus^+^ neurons in naïve, unpaired and paired conditions. Paired training significantly increases the percentage of *Arc*-dVenus^+^ cells compared with naïve and unpaired controls. In contrast, a similar percentage of *Arc*-dVenus^+^ cells is observed between naïve and unpaired conditions (Naïve: *n*=7 mice, Unpaired: *n*=7 mice, Paired: *n*=6 mice). One-way analysis of variance, *F*=8.59, *P<*0.01. (**c**) Cumulative distribution of *Arc*-dVenus fluorescence intensity. Fluorescence intensity is significantly higher in mice receiving paired fear conditioning, compared with naïve and unpaired controls. Kolmogorov-Smirnov: Naïve versus Paired, *D*=0.48, *P<*0.0001; Unpaired versus Paired, *D*=0.24, *P<*0.001. (**d**) Frequency histograms of *Arc*-dVenus fluorescence intensity. *x* axis is truncated at 45 a.u. (Panels **c** and **d**: bin size, 5 a.u.). *P<0.05, **P<0.01, ***P<0.001.

Using confocal stereology, we quantified the percentage and fluorescence intensity of *Arc*-dVenus^**+**^ neurons in the LA following fear conditioning (Figure 2; Supplementary Figure 1). In naïve mice, only weak levels of *Arc*-dVenus fluorescence were detectable in LA neurons, consistent with the low baseline expression of endogenous *Arc.*^47^ Unpaired conditioning did not influence the percentage of *Arc*-dVenus^**+**^ neurons. In contrast, the percentage of *Arc*-dVenus^**+**^ neurons observed in mice receiving paired conditioning was significantly increased compared with naïve (*P*<0.01) and unpaired (*P*<0.05) groups (Figure 2b). Moreover, paired training induced a strong right-shift of the cumulative probability distribution of *Arc*-dVenus fluorescence, compared with naïve (*P*<0.0001) and unpaired (*P*<0.001) conditions (Figure 2c). Together, our findings indicate that the induction of *Arc*-dVenus expression in the LA during fear conditioning is highly specific for associative learning, compared with non-associative sensory stimulation.

To further explore the relationship between the strength of learning, percentage of *Arc*-dVenus^+^ neurons and dVenus fluorescence intensity, we used independent groups of mice trained with 1, 3 or 9 CS-US pairings. Stereological analysis demonstrated an asymptotic percentage of *Arc*-dVenus^+^ neurons beyond 3 CS-US pairings, which closely paralleled the CS-evoked freezing curve (Supplementary Figures 2A and B). Notably, however, despite a similar strength of conditioning and percentage of *Arc*-dVenus^+^ neurons, mice receiving 9 CS-US pairings had a significantly increased dVenus fluorescence intensity compared with mice receiving only 3 CS-US pairings (Supplementary Figures 2C and E). Therefore, successive CS-US pairings do not recruit cells randomly within the LA, but instead result in a highly overlapping re-activation of a similar neuronal subpopulation.

Recent models of memory formation have hypothesized that at any given time, a limited subset of neurons exist in an *a priori* primed state, which could serve to preferentially bias their allocation into a newly encoded associative memory trace.^13, 15, 16, 17, 31, 48, 49^ Therefore, we considered the possible mechanisms by which cellular activation in the LA could transform the *Arc*-dVenus fluorescence intensity curve from the baseline (naïve) state to the distribution observed after fear conditioning (Figure 2c). In particular, the rightward shift in the *Arc*-dVenus fluorescence intensity distribution could have resulted from two non-mutually exclusive possibilities: (i) In Figure 2b, we observed a ~50% increase in the number of *Arc*-dVenus^**+**^ neurons in the LA following paired training. Accordingly, if these newly *Arc*-dVenus^**+**^ neurons are predominantly of high fluorescence intensity, the resulting cumulative probability curve would shift to the right. (ii) A second possibility is that baseline *Arc*-dVenus^**+**^ neurons are preferentially recruited during fear conditioning. Prior to conditioning, ~10% of LA neurons are *Arc*-dVenus^**+**^, and thereby represent the fluorescence intensity distribution of the baseline (naïve) group. During fear conditioning, activation of these baseline *Arc*-dVenus^**+**^ neurons would necessarily increase their fluorescence level and consequently shift the overall population distribution to the right. Therefore, to distinguish between these possibilities, we examined the absolute frequency histograms of fluorescence intensity, which fully account for the difference in the overall percentage of *Arc*-dVenus^**+**^ neurons (Figure 2d). Notably, the fluorescence intensity distribution remained significantly right-shifted despite having fully accounted for the increased percentage of *Arc*-dVenus^**+**^ neurons, and consistent with a model of neuronal selection during fear conditioning in which baseline *Arc*-dVenus^**+**^ neurons are preferentially recruited into the memory trace.

### Preferential recruitment of neurons with enhanced intrinsic excitability

**Figure 3:**
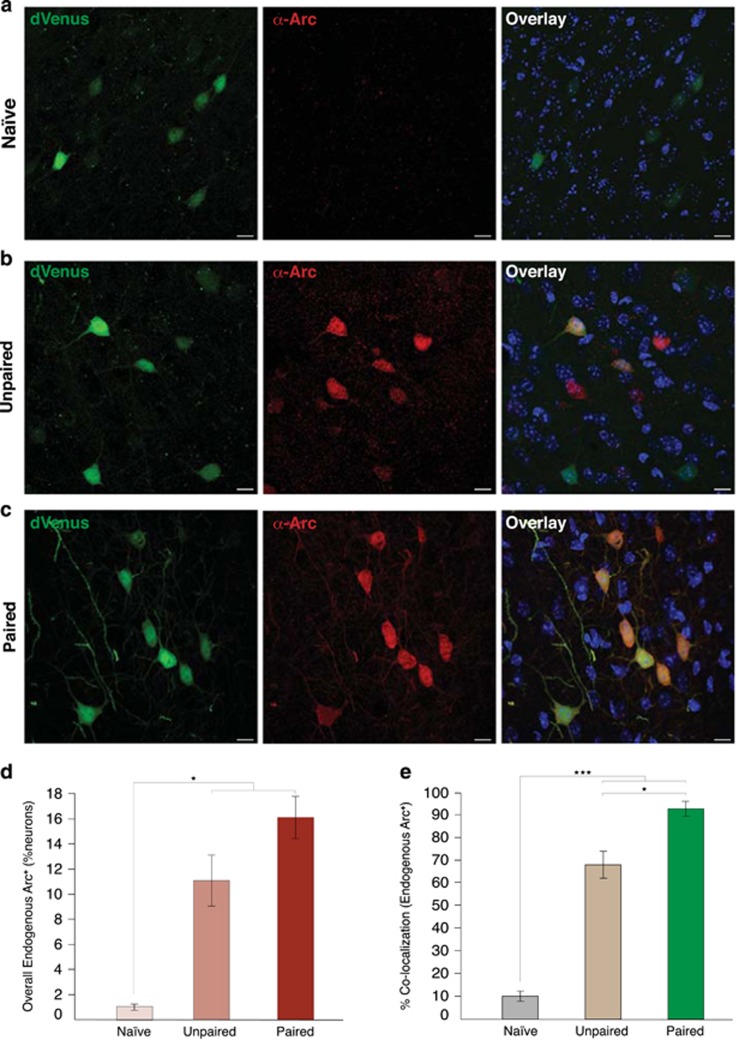
Baseline *Arc*-dVenus^+^ neurons are preferentially recruited during fear conditioning. (**a**–**c**) Representative images of *Arc*-dVenus and endogenous Arc co-localization at 1 h post training. Scale bar, 10 μm. (**d**) Overall percentage of LA neurons expressing endogenous Arc in naïve, unpaired and paired conditions. Paired and unpaired training induce an increase in the number of neurons expressing endogenous Arc. One-way analysis of variance, *F*=25.03, *P<*0.001. (**e**) Endogenous Arc is preferentially localized to *Arc*-dVenus^+^ neurons in mice receiving paired conditioning, compared with naïve or unpaired controls. Two-way analysis of variance, group × *Arc*-dVenus interaction, *F*=94.12, *P<*0.0001. *P<0.05, **P<0.01, ***P<0.001.

To further examine the hypothesis that baseline *Arc*-dVenus^**+**^ neurons are preferentially recruited during fear learning, we made use of the differential half-life of endogenous Arc^50^ compared with dVenus.^32^ Endogenous Arc is nearly undetectable in naïve mice,^47^ and peaks in the LA at 1 h after fear conditioning, specifically marking neurons that were activated during conditioning (Supplementary Figure 3). In contrast, baseline *Arc*-dVenus^**+**^ neurons remain easily detectable over a 1 h period given that the *in vivo* half-life of *Arc*-dVenus fluorescence is 3 h.^32^ Therefore, at 1 h after fear conditioning, a high percentage of *Arc*-dVenus^**+**^ neurons with co-localized expression of endogenous Arc would confirm that baseline *Arc*-dVenus^**+**^ neurons are preferentially recruited during fear conditioning. In contrast, a low rate of co-localized expression would suggest that the baseline *Arc*-dVenus^**+**^ population has no *a priori* bias towards activation. Indeed, consistent with a model of preferential recruitment, 92.6% of *Arc*-dVenus^**+**^ neurons from mice undergoing paired training were co-localized with endogenous Arc, compared with only 10.2% in naïve mice (Figure 3); *P<*0.0001. Mice receiving unpaired training also showed recruitment of baseline *Arc*-dVenus^**+**^ neurons, although the co-localization was significantly lower than in mice receiving paired training (*P<*0.05). Lastly, c-Fos activation was also highly co-localized with *Arc*-dVenus at 1 h post-training (Supplementary Figure 4), demonstrating that this finding is not simply restricted to *Arc*. Together, these data indicate that baseline *Arc*-dVenus expression represents a unique molecular marker for LA neurons that are preferentially recruited during fear memory encoding.

Given that *Arc*-dVenus^**+**^ neurons are preferentially recruited during fear conditioning, their defining electrophysiological properties might offer unique insights into the mechanisms underlying associative memory encoding. Passive membrane properties and single AP characteristics of Arc-dVenus^**+**^ and neighboring *Arc*-dVenus^**−**^ neurons demonstrated no two-way interactions of Arc-dVenus status and training condition (Supplementary Table 1). Furthermore, there were no overall main effects of *Arc*-dVenus status. However, three parameters demonstrated overall main effects of training condition: membrane resistance (*F*_2,104_=5.52, P<0.01), AP threshold (*F*_2,95_=8.37, P<0.001), and AP half-width (*F*_2,95_=6.03, P<0.01) (Supplementary Table 1). *Post hoc* pairwise comparisons across training conditions demonstrated that membrane resistance was significantly lower in mice receiving paired training compared with naïve mice (P<0.01), with no significant differences of either condition in comparison with mice receiving unpaired training. AP threshold was significantly more depolarized in mice from the paired (*P*<0.01) and unpaired (*P*<0.01) condition, compared with naïve mice. Lastly, AP half-width was significantly narrower in mice receiving paired training, compared with naïve (*P*<0.05) or unpaired (*P*<0.01). Importantly, these main effects of training condition are independent of whether the recorded neurons were *Arc*-dVenus^**+**^ or *Arc*-dVenus^**−**^, and therefore reflect global experience-dependent changes observed broadly throughout the LA.

**Figure 4:**
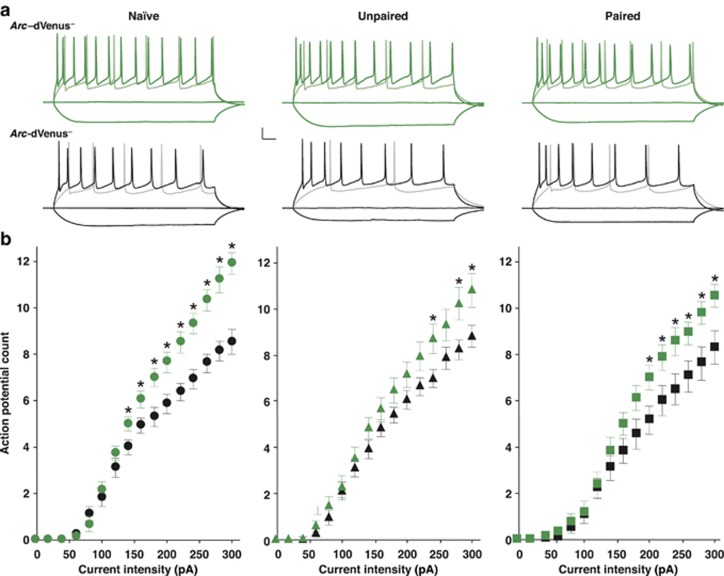
*Arc*-dVenus^+^ neurons exhibit increased excitability. (**a**) Superimposed current-clamp recordings with −100, 0, +140, +300 pA sustained current injection at a holding membrane potential of −75 mV. *Arc*-dVenus^+^ neurons (top) fire more APs than non-activated neighbors (bottom) in naïve, unpaired and paired conditions. Scale bars: 20 mV, 50 ms. (**b**) Plot of the mean AP count versus current injection intensity for naïve (left), unpaired (center) and paired (right) conditions. *Arc*-dVenus^+^ neurons (naïve: *n*=15, unpaired: *n*=17, paired: *n*=16; green) show higher excitability compared with neighboring *Arc*-dVenus^−^ neurons (naïve: *n*=16, unpaired: *n*=17, paired: *n*=18; black). Naïve: *n*=12 mice, Unpaired: *n*=6 mice, Paired: *n*=8 mice. Repeated measures analysis of variance, *Arc*-dVenus × current intensity interaction: naïve, *F*=14.06, *P<*0.001; unpaired, *F*=3.75, *P<*0.05; paired, *F*=3.55, *P<*0.05. *P<0.05.

Intrinsic excitability has been widely hypothesized as a candidate mechanism for neuronal recruitment during associative learning.^15, 16, 17^ However, no previous studies have been able to directly address this hypothesis under entirely physiological conditions. Therefore, we performed targeted whole-cell recordings from *Arc*-dVenus^**+**^ neurons and their non-activated *Arc*-dVenus^**−**^ neighbors. Consistent with the hypothesis that increased excitability might support their preferential recruitment into the fear memory trace, baseline *Arc*-dVenus^**+**^ neurons had significantly higher intrinsic excitability than their non-activated neighbors (Figure 4). Moreover, *Arc*-dVenus^**+**^ neurons from both the paired and unpaired conditions displayed a similar increase in excitability, the magnitude of which was independent of learning or sensory stimulation. Accordingly, *Arc*-dVenus^**+**^ neurons also displayed higher instantaneous AP frequencies than neighboring *Arc*-dVenus^**−**^ neurons (Supplementary Figure 5). Notably however, no differences were observed in AP amplitude or duration across spike trains (Supplementary Figures 6 and 7). Taken together, these findings suggest that enhanced excitability cannot account for the encoding of a fear memory, but rather is highly consistent with a model for neuronal selection during learning regulated by intrinsic excitability.

### Synaptic plasticity is highly localized to *Arc*-dVenus^+^ neurons during fear conditioning, and postsynaptically mediated

**Figure 5:**
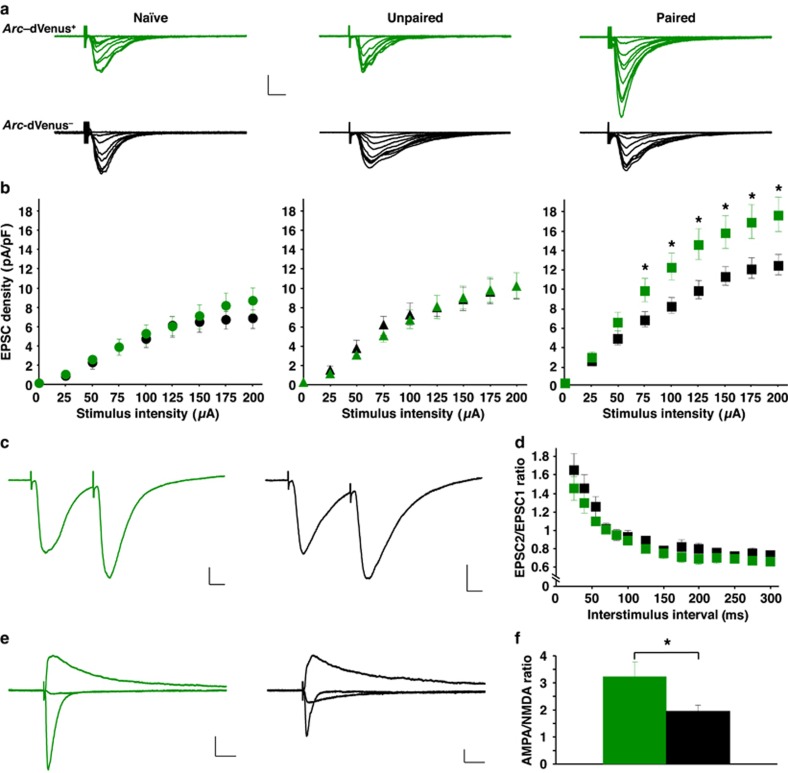
Synaptic potentiation is learning-specific and highly localized to *Arc*^+^ neurons. (**a**) Superimposed averages (5 traces) of EPSCs evoked via thalamic input stimulation (0–200 μA, 25 μA increments) at a holding membrane potential of −70 mV. Stimulus artifacts are truncated. *Arc*-dVenus^+^ neurons (top) display strongly potentiated evoked EPSCs specific to the paired (right) versus naïve (left) or unpaired (center) conditions. Scale bars: 200 pA, 10 ms. (**b**) Input-output curves for naïve (left), unpaired (center) and paired (right) conditions. In the naïve and unpaired conditions, EPSCs recorded from *Arc*-dVenus^+^ (naïve: *n*=13, unpaired: *n*=19; green) and *Arc*-dVenus^−^ (naïve: *n*=14, unpaired: *n*=18; black) neurons are similar. In contrast, EPSCs are selectively potentiated in *Arc*-dVenus^+^ neurons in the paired condition (*n*=34, green) compared with neighboring *Arc*-dVenus^**−**^ neurons (*n*=34, black), across all stimulus intensities >50 μA. Naïve: *n*=12 mice, Unpaired: *n*=12 mice, Paired: *n*=21 mice. Repeated measures analysis of variance, stimulus intensity × *Arc*-dVenus interaction: naïve, *F*=0.88, *P*=0.39; unpaired, *F*=0.28, *P*=0.69; paired, *F*=5.26, *P<*0.01. (**c**) Averages (5 traces) of EPSC pairs (normalized to the first EPSC) with a 45 ms interstimulus interval from *Arc*-dVenus^+^ (left) and *Arc*-dVenus^−^ (right) neurons of mice receiving paired conditioning. Scale bars: 100 pA, 10 ms. (**d**) Paired-pulse ratios were similar between *Arc*-dVenus^+^ (*n*=14, green) and *Arc*-dVenus^−^ (*n*=16, black) neurons from 15 mice. Repeated measures analysis of variance, stimulus intensity × *Arc*-dVenus interaction, *F*=1.08, *P*=0.33. (**e**) Evoked EPSCs (average of 5 traces) at -70, 0 and +40 mV holding membrane potentials, scaled to the +40 mV peak amplitude. *Arc*-dVenus^+^ (left) and *Arc*-dVenus^−^ (right) neurons from mice receiving paired conditioning. Scale bars: 100 pA, 40 ms. (**f**) AMPA/*N*-methyl-_D_-aspartate ratio is significantly increased in *Arc*-dVenus^+^ (*n*=24, green) compared with neighboring *Arc*-dVenus^−^ (*n*=26, black) neurons from 15 mice. *P<0.05.

The encoding of auditory fear memories is thought to occur through selective potentiation of glutamatergic synaptic inputs to the LA.^5, 6, 10, 11, 12^ However, previous studies investigating auditory fear-conditioning-induced synaptic modifications have been performed without knowledge of whether recorded neurons were part of the memory trace. Therefore, the *Arc::dVenus* mice represented a unique opportunity to examine directly whether learning-induced synaptic potentiation is preferentially localized to *Arc*-dVenus^**+**^ neurons, as predicted by a model of sparse memory encoding. We recorded EPSCs evoked by stimulation of thalamic afferents to LA neurons. In both naïve and unpaired conditions, similar EPSC amplitudes were observed in *Arc*-dVenus^**+**^ and neighboring *Arc*-dVenus^**−**^ neurons (Figures 5a and b). In contrast, paired conditioning induced a robust and highly specific potentiation of thalamic afferent synapses, selectively in *Arc*-dVenus^**+**^ neurons (Figures 5a and b). Therefore, *Arc* expression defines the LA neuronal ensemble onto which synaptic plasticity is highly localized during fear conditioning.

We next sought to determine whether the site of plasticity for the enhancement in glutamatergic synaptic transmission during auditory fear conditioning was pre- or postsynaptic. If fear conditioning differentially modifies the neurotransmitter release probability onto *Arc*-dVenus^**+**^ versus *Arc*-dVenus^**−**^ neurons, such a change should be evident by a decrease in the paired-pulse ratio for glutamatergic inputs onto *Arc*-dVenus^**+**^ neurons compared with neighboring *Arc*-dVenus^**−**^ neurons. Therefore, we performed paired-pulse stimulation of thalamic afferents across a range of interstimulus intervals from 25 to 300 ms (Figures 5c and d). Notably, *Arc*-dVenus^**+**^ and *Arc*-dVenus^**−**^ neurons showed similar paired-pulse ratios across all interstimulus intervals examined, making it unlikely that the learning-induced synaptic potentiation of *Arc*-dVenus^**+**^ neurons was presynaptic in origin.

Alternatively, we measured the ratio of AMPA to NMDA currents, a widely used measure that is highly sensitive to postsynaptically mediated plasticity of glutamatergic transmission, including long-term potentiation.^6, 51^ Indeed, consistent with a postsynaptic locus of plasticity, fear conditioning induced a significant increase in the AMPA/NMDA current ratio in *Arc*-dVenus^**+**^ neurons compared with their *Arc*-dVenus^**−**^ neighbors (Figures 5e and f). Taken together, our findings demonstrate that learning-induced synaptic potentiation is postsynaptically mediated and selectively localized onto the sparse population of *Arc*-expressing neurons.

## Discussion

The elucidation of the physiological mechanisms underlying memory encoding remains a considerable technical challenge, owing to the sparseness of neuronal representations. Therefore, we used a novel *Arc* reporter mouse^32, 45, 52^ to permit visual identification and neurophysiological interrogation of neurons with recent activation. Using this powerful approach for exploring learning-specific alterations in neuronal physiology, we now demonstrate that fear-conditioning-induced glutamatergic synaptic potentiation in the LA is preferentially localized to *Arc*^**+**^ neurons, thereby confirming the sparse encoding hypothesis and identifying *Arc* as a *bona fide* molecular marker of the LA fear memory trace. Furthermore, we show that baseline differences in neuronal excitability are highly predictive of the ensemble of neurons selectively recruited into the fear memory trace.

We found that the potentiation of glutamatergic synaptic transmission from the thalamic input pathway was postsynaptically mediated, given the highly significant enhancement in AMPA/NMDA ratio from mice receiving paired training, in the absence of changes in the presynaptically mediated paired-pulse ratio. These findings are consistent with the comprehensive series of previous studies reporting a postsynaptically mediated plasticity of the thalamic input pathway,^5, 6, 9, 53^ although a minority of reports have also suggested the contribution of a presynaptic mechanism.^54^ Nonaka *et al.*^52^ recently used the *Arc::dVenus* mice to examine neuronal recruitment and synaptic plasticity following contextual conditioning in the basolateral amygdala. Similar to our findings, they observed a preferential recruitment of *Arc*-dVenus^**+**^ neurons evident in both the learning and non-associative conditions. Moreover, a presynaptically mediated potentiation of cortical-basolateral amygdala synaptic transmission was observed selectively in Arc-dVenus^**+**^ neurons, as evidenced by an increase in mEPSC frequency and a decrease in paired-pulse ratio. Taken together, these findings demonstrate that the induction of Arc IEG activation is a highly reliable marker for identifying the limited subset of neurons recruited to the fear memory trace and defined by pathway-specific alterations in synaptic transmission.

Previous studies demonstrating postsynaptically mediated plasticity of the thalamic input pathway to the LA using whole-cell patch-clamp recordings were performed in randomly chosen LA neurons without knowledge of their *Arc* expression.^5, 6^ Our findings now extend these results by demonstrating that potentiation of glutamatergic synaptic transmission occurs disproportionately onto *Arc*^+^ neurons. However, this also raises an important question regarding the *Arc*^**−**^ population, which presumably constitute a substantial proportion of the recorded neurons. Notably, although not statistically significant, there was a strong trend for increased synaptic transmission within the *Arc*^**−**^ population in mice receiving paired training, compared with the unpaired and naïve groups (Figure 5b). Moreover, there are two notable aspects of our experimental design that may also be important to consider. First, we performed the electrophysiological recordings directly following training without intervening memory testing, given the increasing literature demonstrating that retrieval of newly learned associations modifies synaptic physiology.^12, 55, 56, 57, 58^ Given the highly divergent CS-evoked freezing responses between the paired and unpaired groups, electrophysiological recordings would have always been confounded by the impact of their differential fear responses during the intervening test session.

Second, we chose a behavioral training protocol that did not result in overtraining (Supplementary Figure 2). In contrast, previous studies of thalamic-LA synaptic transmission demonstrating postsynaptically mediated plasticity have used stronger conditioning protocols. With more robust training, it is possible that changes in thalamic-LA synaptic transmission might have occurred outside the *Arc*^+^ population. Alternatively, in the setting of more robust training the *Arc*^+^ population might have constituted a significantly larger proportion of LA neurons than we have observed, for which random sampling would have yielded the previously reported effects yet consistent with learning-specific changes in thalamic-LA synaptic transmission being largely restricted to the Arc^+^ population. Importantly however, this latter possibility is inconsistent with our finding of an asymptotic percentage of Arc^+^ neurons beyond 3 CS-US pairings.

In addition to learning-induced plasticity, we also observed that neurons with baseline elevation of intrinsic excitability are preferentially recruited into the fear memory trace. Previous studies using viral-mediated overexpression of CREB have proposed intrinsic excitability as a candidate cellular mechanism for neuronal selection during fear learning in the LA.^15–17,59^ However, it remained unknown to what extent these findings recapitulated the endogenous physiological mechanism. Our present results add compelling evidence that intrinsic excitability is indeed a highly influential cellular mechanism underlying recruitment of individual LA neurons during fear memory encoding. Interestingly, studies in both vertebrate and invertebrate species have demonstrated robust and enduring learning-induced alterations in neuronal excitability.^60, 61, 62, 63, 64, 65, 66, 67^ Our findings provide strong support for the hypothesis that neurons with baseline elevation of intrinsic excitability are preferentially recruited into the memory trace, and may serve to bind together experiences acquired closely together in time. Moreover, we speculate that the recent elegant studies using targeted manipulations of IEG-defined memory traces are influencing neuronal selection during learning precisely through modulation of neuronal excitability.^30, 31^

We observed an asymptotic percentage of *Arc*-dVenus^+^ neurons beyond 3 CS-US pairings, despite a further increase in dVenus fluorescence intensity in mice receiving 9 CS-US pairings. Therefore, neuronal selection during fear learning appears to be constrained by the intrinsic microcircuitry of the LA, leading to re-activation of a similar neuronal subpopulation upon successive CS-US pairings throughout the training session. Given the extensive inhibitory network within the LA, a feed-forward inhibitory microcircuitry is a well-suited candidate for mediating this outcome.^68, 69^ Previous studies have demonstrated unique mechanisms of inhibitory interneuron plasticity within the LA that are likely to function critically in both neuronal selection and fear memory encoding.^17, 68, 69, 70, 71, 72^ Future studies using multicellular recordings will be required to more precisely define the local microcircuit connectivity and cell-type-specific mechanisms of plasticity.

Our experiments utilized two independent control groups: (i) Unpaired: mice receiving explicitly unpaired CS and US presentations but matched with the paired condition regarding the number and specifications of the CS and US, context exposure and handling; and (ii) Naïve: mice that were truly naïve to any experimental manipulations in that they had no context exposures, nor any handling beyond their standard housing conditions. Our rationale for this design was that the unpaired condition would provide the ideal control for handling, context exposure and the influence of CS and US stimuli independent of auditory fear conditioning. However, any differences observed between the naïve and unpaired groups remain difficult to precisely attribute etiologically, as these effects could be due to the handling, context exposure, CS and/or US stimuli. Moreover, mice receiving unpaired training undergo contextual conditioning. Differences in the naïve and unpaired groups were observed exclusively in experiments examining dVenus fluorescence intensity (Figures 2c and d) and neuronal recruitment (Figure 3), in which the effect size was, in both cases, smaller than observed in the paired condition. No differences were observed in electrophysiological recordings comparing dVenus^+^ and dVenus^**−**^ neurons between the naïve and unpaired conditions. Rather, the only electrophysiological difference observed between naïve and unpaired mice regarded AP threshold (Supplementary Table 1), an effect that was independent of dVenus status. Therefore, learning-specific changes in synaptic plasticity cannot be accounted for by handling, context exposure or the unpaired presentation of CS and US stimuli. In contrast, neuronal recruitment in the LA appears to occur independently of auditory fear learning, and mediated by one or more of the stimuli distinguishing the unpaired and naïve groups. Given the function of the LA in assigning emotional valence, we would hypothesize that the strong recruitment in the unpaired condition likely results from the stress sensitization of the US stimuli, but future studies will be required to examine this in further detail.

Taken together with previous findings, we propose a model of fear learning in which non-associative neuronal selection and Hebbian synaptic encoding of the learned association are distinct physiological processes: intrinsic excitability determines neuronal selection, whereas learning-related encoding is governed by synaptic plasticity.
