# Supplementary figures and images for: Arc expression identifies the lateral amygdala fear memory trace
Source: Mol Psychiatry. 2015 Mar 24;21(3):364–75. doi: 10.1038/mp.2015.18 (PMC4759206; doi:10.1038/mp.2015.18)

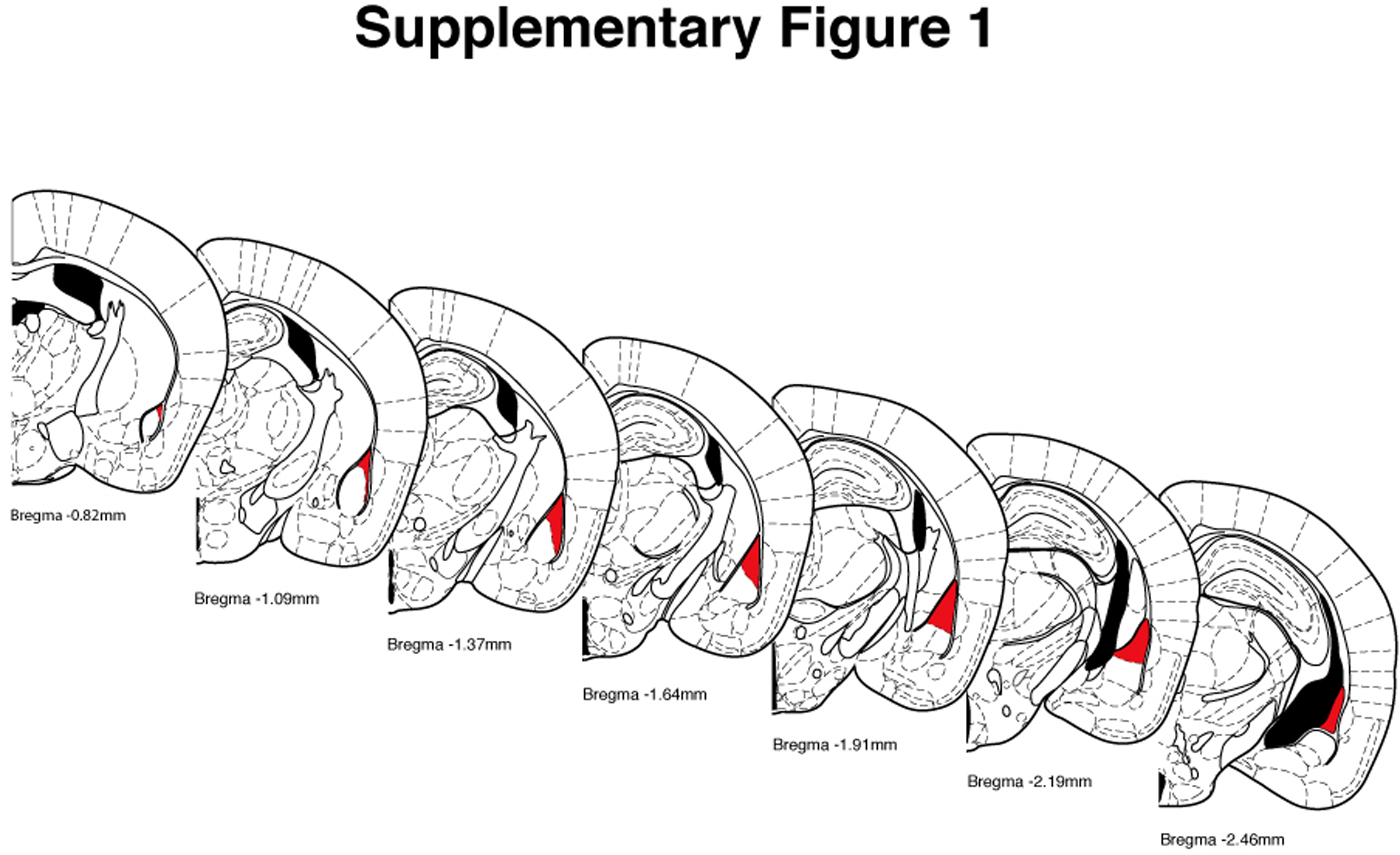

Supplement: Supplementary Figure 1 [file mp201518x1.tif]

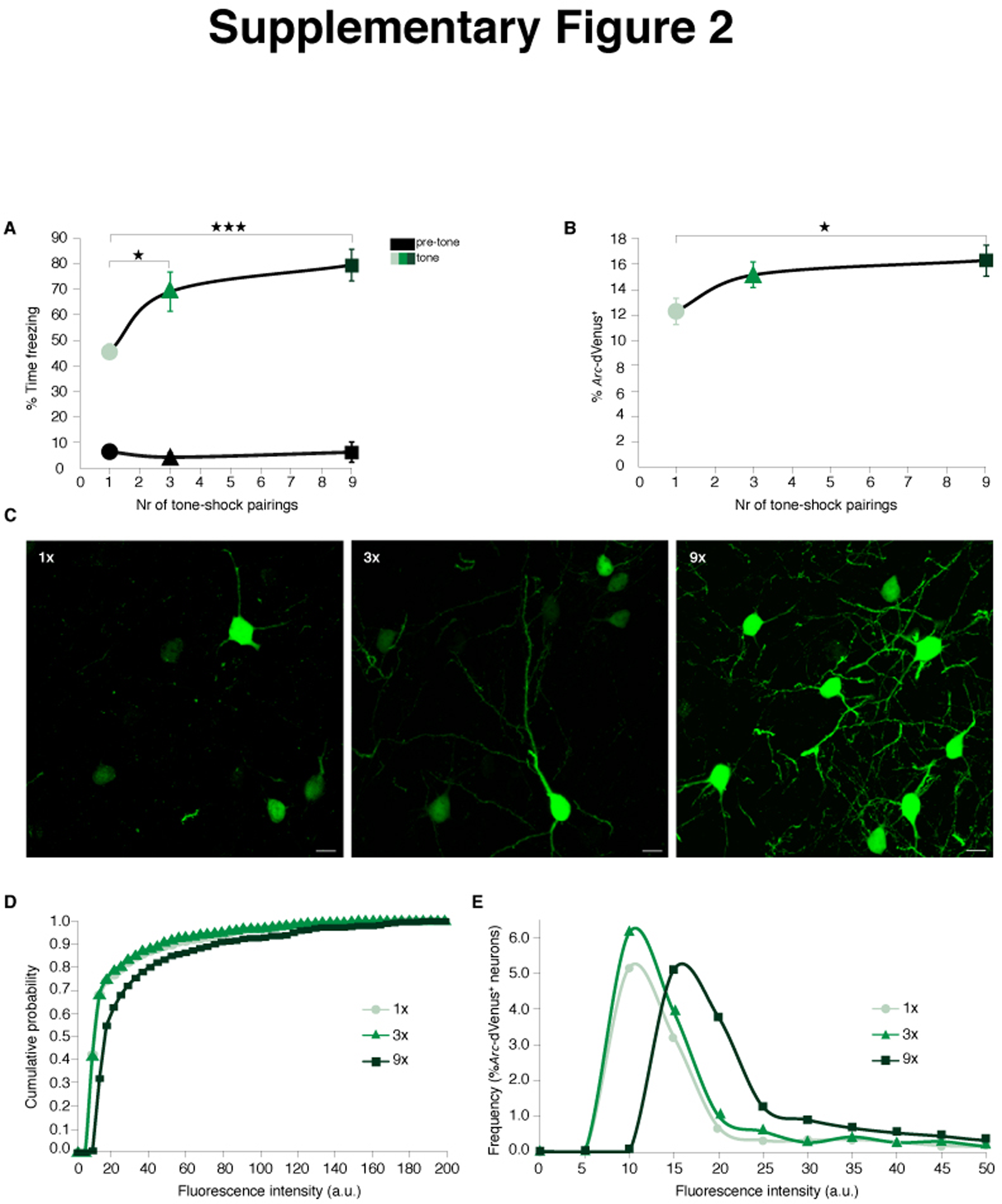

Supplement: Supplementary Figure 2 [file mp201518x2.tif]

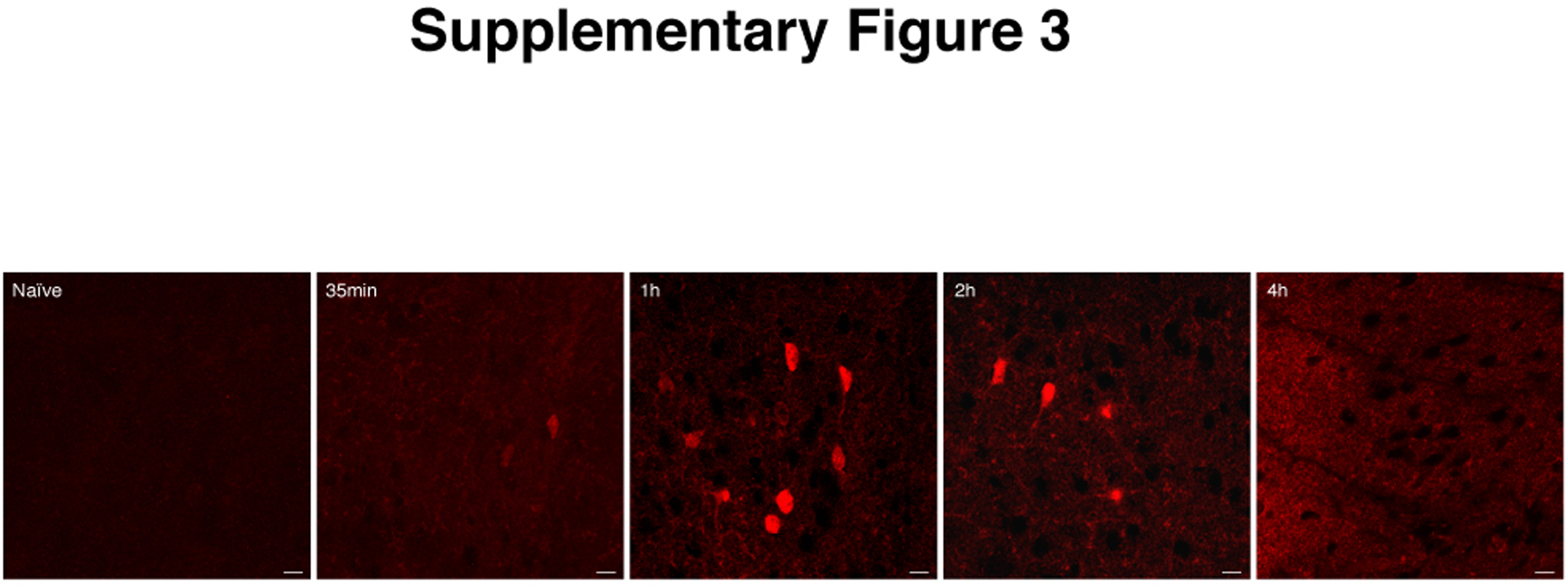

Supplement: Supplementary Figure 3 [file mp201518x3.tif]

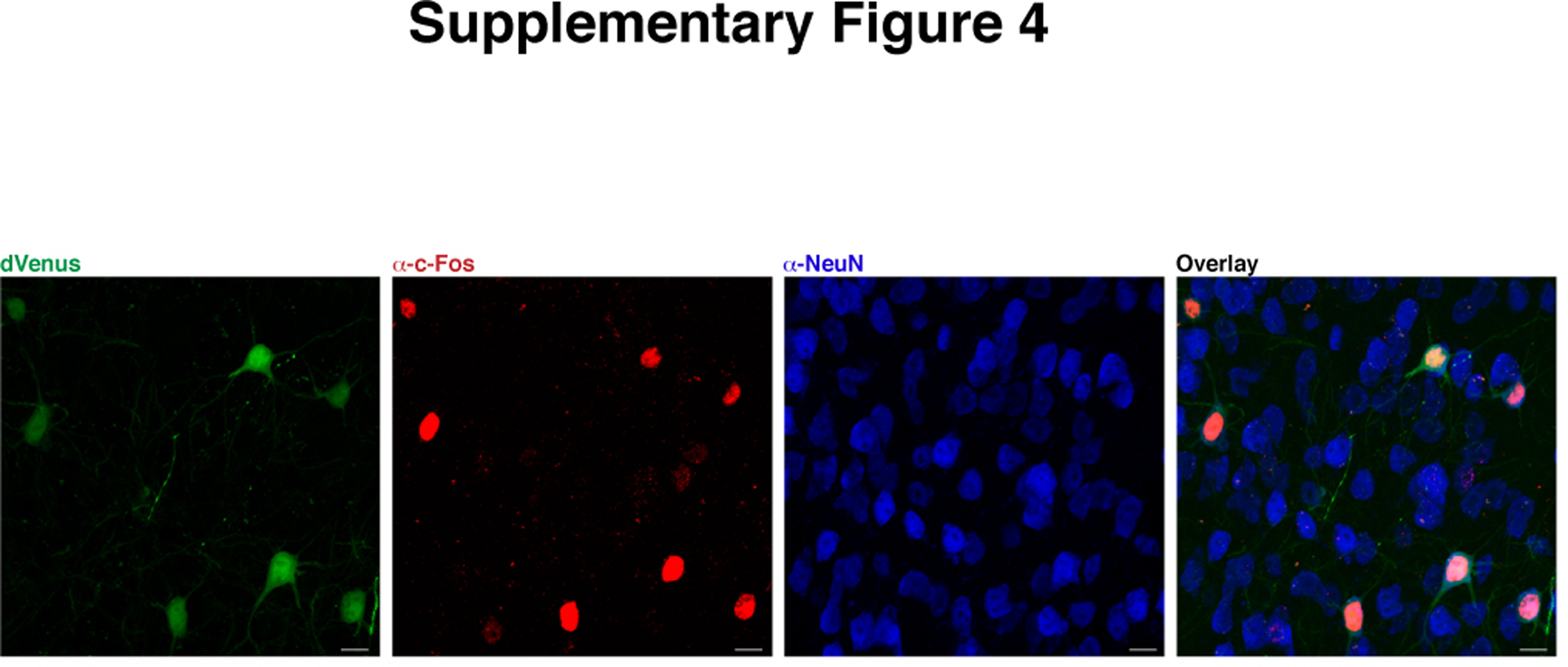

Supplement: Supplementary Figure 4 [file mp201518x4.tif]

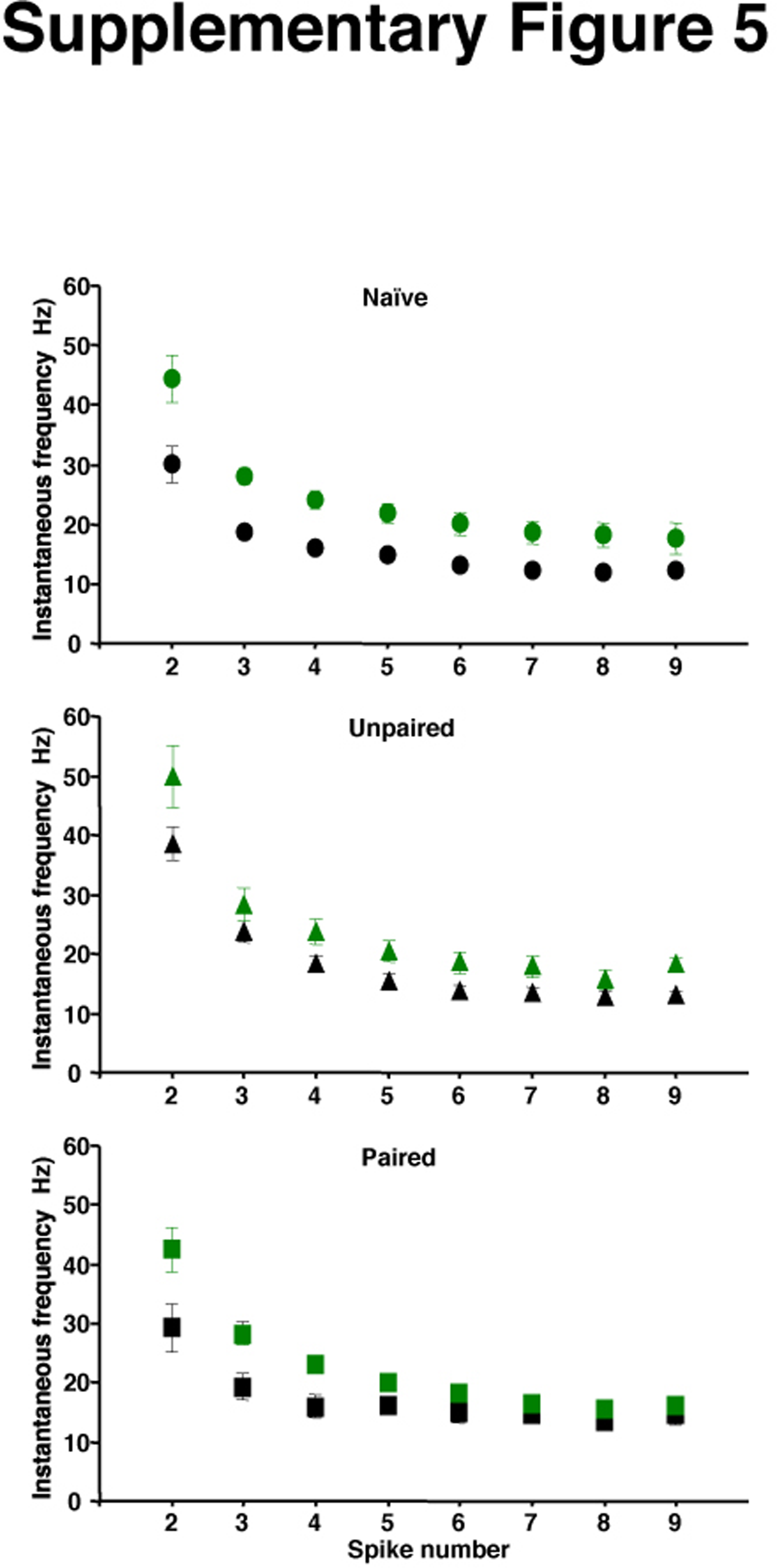

Supplement: Supplementary Figure 5 [file mp201518x5.tif]

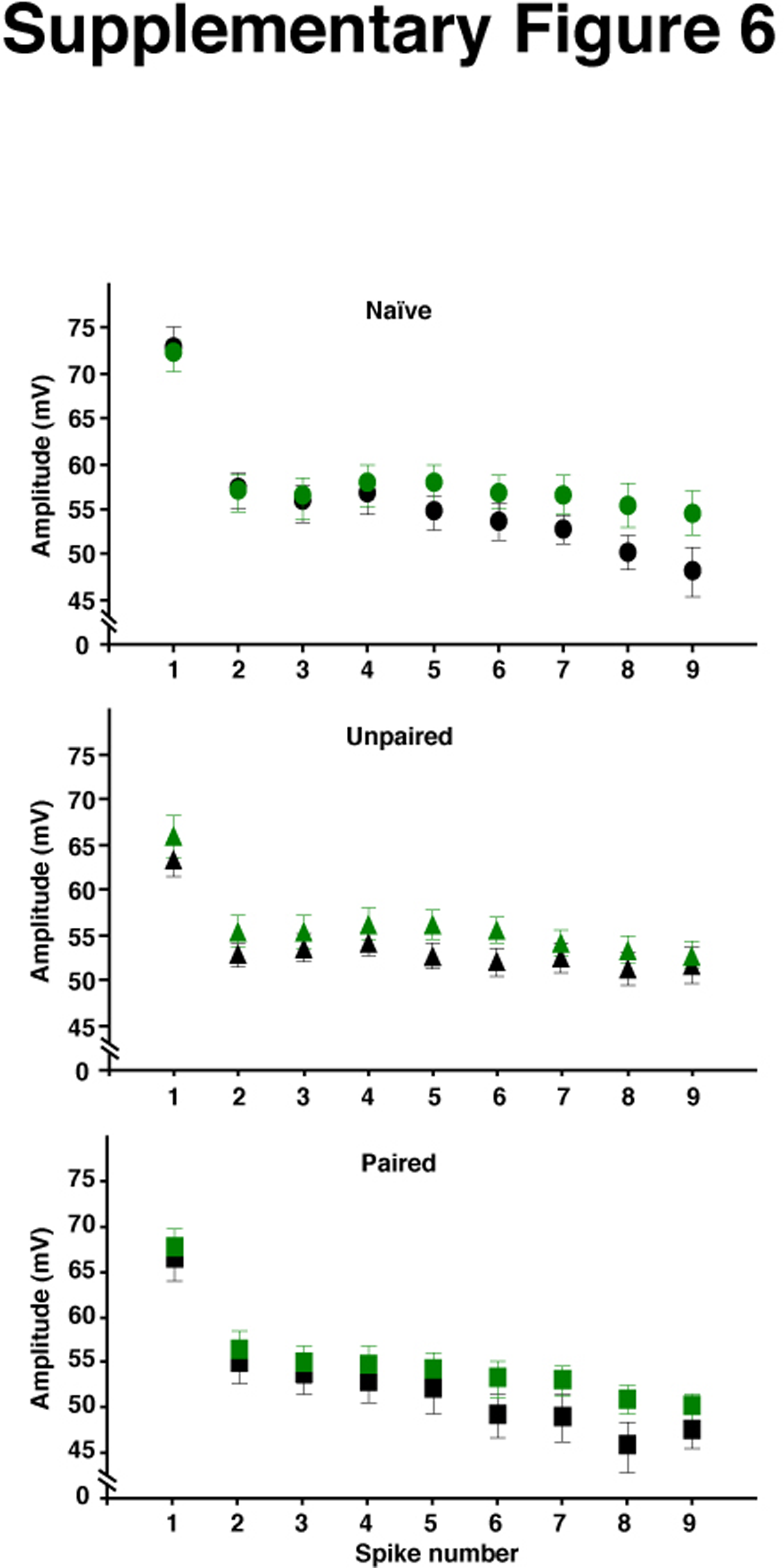

Supplement: Supplementary Figure 6 [file mp201518x6.tif]

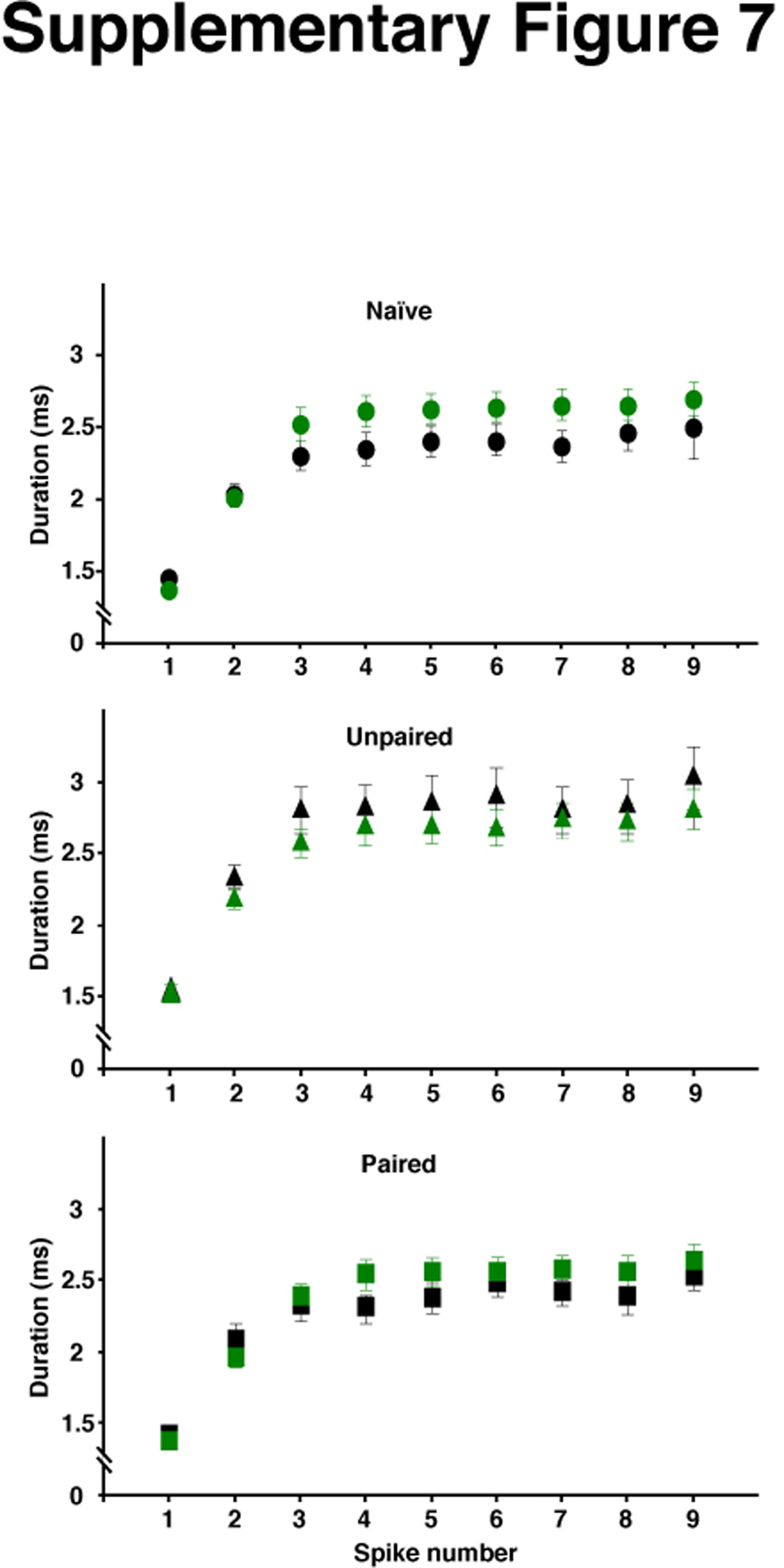

Supplement: Supplementary Figure 7 [file mp201518x7.tif]

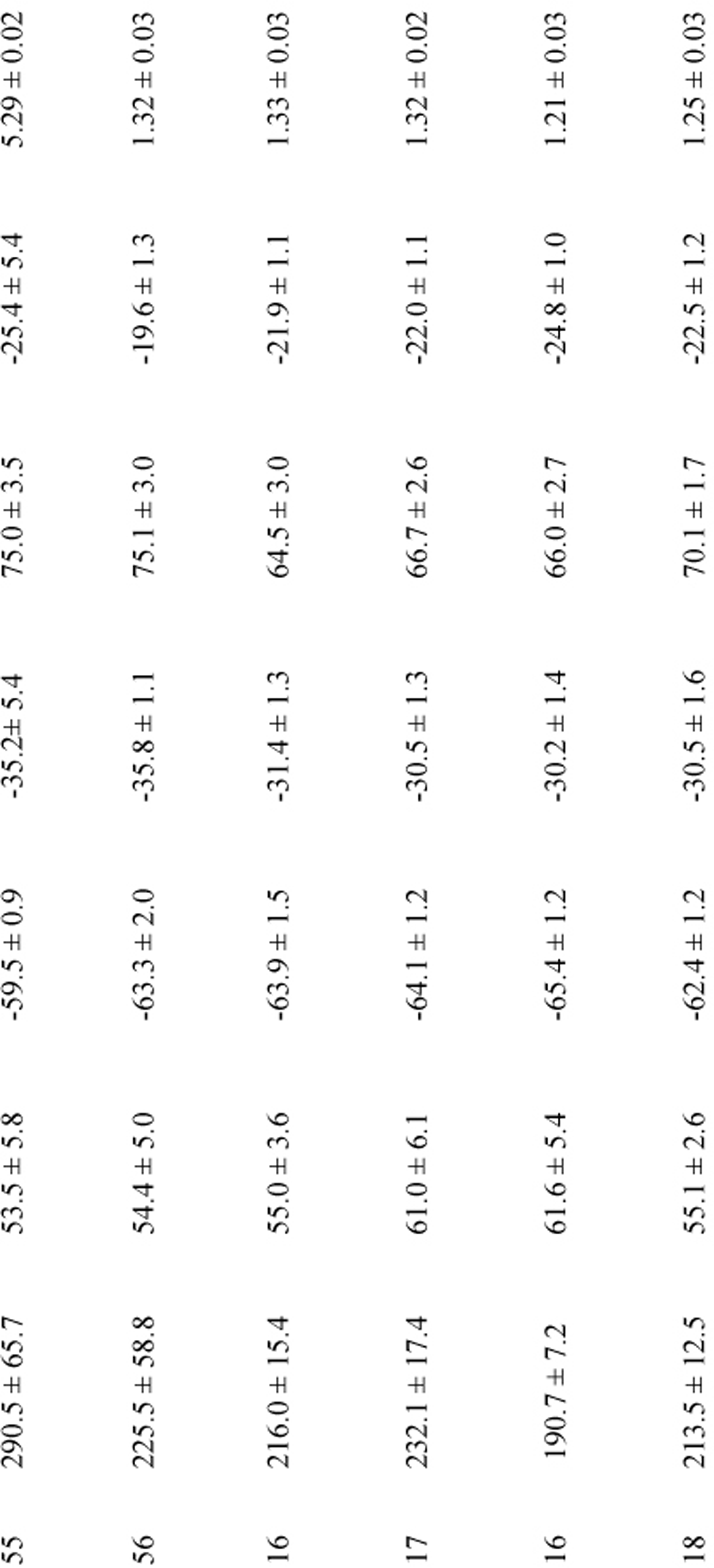

Supplement: Supplementary Table 1 [file mp201518x8.tif]
