# Supplementary material for: Arc expression identifies the lateral amygdala fear memory trace
Source: Mol Psychiatry. 2015 Mar 24;21(3):364–75. doi: 10.1038/mp.2015.18 (PMC4759206; doi:10.1038/mp.2015.18)
Supplement: Supplementary Figure and Table Legends [file mp201518x9.doc]

**Supplementary Figure/Table Legends**

**Supplementary Figure 1: Serial sections of LA for confocal stereology.** Stereological analysis was performed systematically at 160 µm intervals across the entire rostro-caudal extent of the LA using coronal brain sections (red). Bregma coordinates are shown based upon the *in vivo* grid, prior to tissue processing. Modified from Paxinos and Franklin^1^.

***Supplementary Figure 2: Influence of the strength of conditioning on Arc-dVenus expression. (A)*** *Tone-induced freezing in mice trained with 1, 3, or 9 CS-US pairings (Naïve: n=8 mice, Unpaired: n=7 mice, Paired: n=8 mice). One-way ANOVA, F=9.34, P<0.001.* ***(B)*** *Stereological quantification of Arc-dVenus****^+^*** *neurons (Naïve: n=6 mice, Unpaired: n=6 mice, Paired: n=6 mice). One-way ANOVA, F=3.65, P<0.05.* ***(C)*** *Native dVenus fluorescence at 5h post-training. Scale bar, 10 µm.* ***(D)*** *Cumulative distribution of Arc-dVenus fluorescence intensity. Fluorescence intensity is significantly higher in mice receiving 9 CS-US pairings, compared to mice receiving only 1 or 3. Kolmogorov-Smirnov: 1 vs. 9, D=0.23, P<0.01; 3 vs. 9, D=0.25, P<0.01.* ***(E)*** *Frequency histograms of Arc-dVenus fluorescence intensity. X-axis is truncated at 50 a.u. (Panels D and E: bin size, 5 a.u.). *P<0.05, ***P<0.001*

***Supplementary Figure 3*: Time course of endogenous Arc activation in the LA following fear conditioning.** Representative images of endogenous Arc expression at multiple time points after paired conditioning. Endogenous Arc expression is higher at 1h after fear conditioning compared to other time points and to naïve mice. Scale bar, 10 µm.

***Supplementary Figure 4*: Co-localization of *Arc*-dVenus and endogenous c-Fos.** Endogenous c-Fos and *Arc*-dVenus expression are highly co-localized in the LA at 1h following paired conditioning. Scale bar, 10 µm.

***Supplementary Figure 5*: *Arc*-dVenus^+^ neurons display higher instantaneous AP frequencies during spike trains.** Plot of the mean AP instantaneous frequencies from the maximal number of evoked AP for naïve (top), unpaired (middle) and paired (bottom) conditions. *Arc*-dVenus**^+^** neurons (naïve: *n=*15, unpaired: *n=*17, paired: *n=*16; filled symbols) show higher AP instantaneous frequencies compared to neighboring *Arc*-dVenus**^–^** neurons (naïve: *n=*16, unpaired: *n=*17, paired: *n=*18; open symbols). *Naïve: n=12 mice, Unpaired: n=6 mice, Paired: n=8 mice*. **P <* 0.05.

***Supplementary Figure 6*: *Arc*-dVenus^+^ neurons have the same AP amplitude during spike trains.** Plot of the mean AP amplitude from the maximal number of evoked AP for naïve (top), unpaired (middle) and paired (bottom) conditions. *Arc*-dVenus**^+^** neurons (naïve: *n=*15, unpaired: *n=*17, paired: *n=*16; filled symbols) and *Arc*-dVenus**^–^** neighbors (naïve: *n=*16, unpaired: *n=*17, paired: *n=*18; open symbols) have similar AP amplitude. *Naïve: n=12 mice, Unpaired: n=6 mice, Paired: n=8 mice*.

***Supplementary Figure 7*: *Arc*-dVenus^+^ neurons have similar AP duration during spike trains.** Plot of the mean AP half-width from the maximal number of evoked AP for naïve (top), unpaired (middle) and paired (bottom) conditions. *Arc*-dVenus**^+^** neurons (naïve: *n=*15, unpaired: n=17, paired: n=16; filled symbols) and *Arc*-dVenus**^–^** neighbors (naïve: *n=*16, unpaired: n=17, paired: n=18; open symbols) have the same AP duration. *Naïve: n=12 mice, Unpaired: n=6 mice, Paired: n=8 mice*.

**Supplementary Table 1: Passive membrane properties and single AP characteristics.** *Arc*-dVenus**^+^** and *Arc*-dVenus**^–^** neurons displayed similar passive and active membrane properties in naïve, unpaired and paired conditions. *Naïve: n=12 mice, Unpaired: n=6 mice, Paired: n=7 mice*.

^1^Paxinos, G. & Franklin, K. B. J. *The Mouse Brain in Stereotaxic Coordinates, Deluxe 2nd edition*. (Academic Press, 2001).
